# Teaching at scale: a mixed-methods study of team-based learning analytics to enable early support in medical education

**DOI:** 10.1186/s12909-026-09612-x

**Published:** 2026-06-15

**Authors:** Elizabeth Sheader, Lisa Donlan, Connor Allen, Nathan Betteridge, Michael Dixon, Ingrid Gouldsborough, Ruth Grady, Margaret Kingston, Valerie Kouskoff, Kerry Parris, Tristan Pocock, Maiedha Raza, Paul Shore, Nicholas Stafford, Cathy Tournier, Michelle Webb, Michael P. Smith

**Affiliations:** 1 Stopford Building, Oxford Road, Manchester, M13 9PL England; 2 Simon Building, Brunswick Street, M13 9PS Manchester, England; 3 Michael Smith Building, Dover Street, Manchester, M13 9NT England

**Keywords:** Medical Curriculum Reform, Team-Based Learning, Learning Analytics, Early Intervention, At-Risk Students

## Abstract

**Background:**

Medical schools are under increasing pressure to teach larger cohorts while maintaining an excellent student experience. The University of Manchester replaced Problem-Based Learning (PBL) with Team-Based Learning (TBL) in 2023 to meet growing demand for medical education. While the educational effectiveness of TBL is well established, this study evaluated how the routine data generated within large-scale TBL could be potentially used to support early intervention, monitor curriculum quality, and validate peer assessment processes.

**Methods:**

We conducted a prospective mixed-methods evaluation of two consecutive Year-1 medical cohorts at a large UK medical school (2023–24, *n* = 435; 2024–25, *n* = 426). Data included TBL individual readiness assurance test (iRAT) scores, student experience surveys, operational measures, and end-of-semester examination results. In the second year, the 50 lowest-performing students after four teaching themes were identified using iRAT data and offered tailored academic and wellbeing support through their academic advisors.

**Results:**

TBL maintained high student satisfaction (74% rated sessions “excellent”) whilst simultaneously reducing weekly staff facilitator hours by 63% (160 h vs. 59 h) and room use by 84% (80 h of bookings vs. 13). Student individual weekly performance, as measured by iRAT scores, was strongly predictive of summative performance (R²≈0.38–0.41) and this finding is reproducible over consecutive years. Theme-level variation in iRAT performance also identified differences in content difficulty and alignment, providing a programme-level indicator of curriculum quality and areas of weaker delivery. Early identification and intervention of struggling students increased semester performance by 20% (*p* < 0.0001) among the bottom 50 students, improved summative exam marks by 5%, and reduced exam failure rate by 41%, without inflating whole cohort attainment.

**Conclusions:**

This study shows how routine TBL data can function as a scalable analytics system to support early intervention, continuous curriculum monitoring, and quality assurance of peer assessment, while also reducing operational demands. These findings provide a transferable model for institutions seeking to expand capacity while maintaining educational quality and equity.

**Supplementary Information:**

The online version contains supplementary material available at https://doi.org/10.1186/s12909-026-09612-x.

## Background

Medical education is navigating simultaneous pressures of an increasing need to educate at scale, challenges in maintaining equity, and accelerating changes in clinical practice [1–4]. These challenges are not unique to a single institution. Expansion of medical school places, increasing class sizes, and workforce shortages have created a shared international need for teaching models that are scalable, resource-efficient, and capable of supporting diverse learners. As of 2025, the University of Manchester (UoM) is the largest medical school in the United Kingdom [5], with approximately 3,100 undergraduate medical students across all years. As such, the programme provides a useful large-scale test case for approaches intended to address challenges faced across medical schools.

In 2023, Problem-Based Learning (PBL) was replaced with Team‑Based Learning (TBL) as the dominant pedagogy in Years 1–2 of the UoM MBChB programme. TBL offers a structured alternative way of teaching that is designed to promote individual accountability, collaborative reasoning, and reliable delivery at scale [6–8]. The aims of this curriculum change were to improve the consistency of teaching, ease staffing and classroom allocation pressures, and address sector-wide concerns about differential attainment relating to well-documented widening participation and ethnicity‑related awarding gaps [3, 9, 10].

One of the key advantages of TBL is the generation of frequent, low-stakes performance data through readiness assurance testing and peer evaluation. Although these data have clear potential to support early identification of struggling students, their analytic value has rarely been demonstrated at scale in medical education. At programme level, such metrics may support three functions: (1) early identification of students at risk of under-performance, (2) continuous monitoring of curriculum alignment and content difficulty, and (3) evaluation of the validity of behavioural assessments such as peer review. Evidence demonstrating these uses at scale in operational medical programmes remains limited. This study therefore evaluates whether large-scale implementation of TBL can maintain student experience, reduce operational burden, and provide reliable early indicators capable of informing personalised intervention for students at risk of underperformance.

### The transition from PBL to TBL for a large medical programme

UoM introduced a fully integrated PBL curriculum in 1994 [11, 12]; the aim was to shift the focus from passive, didactive teaching to student-centred problem‑first enquiry and self-directed learning [13, 14]: this model is now delivered in a hybrid style in the majority of medical universities in the UK [15]. However, over the past 25 years, expectations of medical graduates have expanded to include far broader knowledge and competencies [16]. Additionally, we are in a period of proposed major structural changes to the NHS workforce. The *NHS Long Term Workforce Plan* [17] sets an ambition to double medical school places in England to 15,000 per year by 2031/32 [18, 19].

The alignment between PBL’s original design and contemporary competency demands has become increasingly weak when teaching large scale cohorts. Multiple reviews have questioned the capacity of PBL to consistently cultivate and/or assess necessary skills, particularly when implemented at scale [20–22]. Studies highlight that the pedagogy is highly dependent on tutor skill and group dynamics, leading to inconsistent development of necessary professional competencies in large cohorts, while also being resource‑intensive and difficult to sustain in very large cohort sizes [20–25]. These conditions led to exploring TBL as a structure capable of reconciling scale with consistency and equity of education.

TBL is an evidence based, active learning pedagogy that was specifically designed to promote both individual accountability and collaborative problem-solving within large cohorts [6, 7, 26]. Unlike traditional PBL, which relies on open ended discussion, TBL employs a fixed and specific sequence of activities [27]. Ahead of time, students first complete pre-work (which can take various guises). Then, during their TBL session, students undertake an individual readiness assurance test (iRAT), which consists of multiple-choice questions that students answer under exam conditions. Immediately following this, students complete the team readiness assurance test (tRAT), which consists of the same multiple-choice questions again, but this time the students must debate the correct answer in their team, and they receive immediate feedback on their responses. Together, the iRAT and the tRAT confirm and refine understanding of core concepts, which are then applied to authentic, clinically relevant problems in a series of Application Exercises that form the central component of the TBL session. Finally, students are asked regularly to peer review the contributions and efforts of their fellow team members, ensuring that students are accountable to their teams.

This pedagogy emphasises the active application of knowledge, teamwork, leadership, and peer learning, while also allowing for immediate formative feedback. Numerous reviews have consistently demonstrated that TBL enhances engagement, promotes deeper learning, and improves knowledge retention, while also maintaining scalability for large cohorts [28–32]. TBL’s structured cycle of activities (prework, iRAT, tRAT, AEs, Peer-Review) serves as a template that mitigates staff variability [31, 33–35]. The educational effectiveness of TBL has been well demonstrated. However, far less attention has been paid to how the rich performance data generated within TBL can be used to inform programme-level decision-making.

TBL was originated by Larry Michaelsen in business education in the late 1970s [6)] However, the growing need within medical education for a structured form of teaching that addresses the need for scalable, collaborative learning without intensive staffing requirements has led to the pedagogy becoming increasingly popular [31, 34, 36, 37]. Within medicine, TBL was first adopted in the United States [38, 39] and the pedagogy subsequently gained traction in Australia, demonstrating TBL’s scalability and effectiveness in large cohorts [26]. The UK has been slower to adopt TBL, with early uptake concentrated in pharmacy education, spearheaded by the University of Bradford [40–42], rather than medicine. Building on this foundation, UoM became the first large UK medical school to transition from an established teaching delivery (PBL) to TBL.

A key challenge in large programmes is the timely identification and support of students at risk of under‑attainment. Traditional PBL implementations often rely on infrequent major assessments and variable tutor judgements of contributions/understanding, limiting opportunities for early intervention [20]. By contrast, TBL generates regular, reliable data on student performance and engagement through iRAT scores and peer evaluation. Within a programmatic‑assessment paradigm, these data may serve as early indicators for personalised intervention [43, 44]. We sought to quantify whether TBL-derived analytics predict performance and whether bespoke support, triggered by early, regular, and cumulative performance indicators, improves attainment for under-performing students.

### Aims

Specifically, we use a mixed-methods approach, consisting of statistical analysis of iRAT and examination data, student experience survey responses, and operational resource data, to explore three interrelated research questions:

1. We investigated whether iRAT performance varies meaningfully across the themed medical weeks that structure the curriculum and if it was robust to environmental factors such as time of day and room size. This approach allowed us to assess whether readiness metrics reflect genuine learning differences rather than logistical variables.
2. We tested the predictive validity of these readiness measures by evaluating the extent to which iRAT scores predict end-of-semester examination performance, and whether data from the first four weeks of learning can identify struggling students who needed interventions.
3. We assessed whether analytics-informed, bespoke interventions produce measurable improvements in readiness and summative performance among at-risk learners.

Collectively, these analyses were designed to evaluate whether TBL-derived data could function not only as student assessment, but as a scalable programme-level analytics system supporting early intervention, curriculum quality assurance, and validation of behavioural measures in large medical programmes.

## Methods

### Setting and participants

Each week began with a mandatory whole-cohort session introducing the clinical theme through interactive activities. This opening session represented one of two significant additions that UoM incorporated into the traditional TBL model. Throughout Monday–Thursday students attended lectures, clinical consultation skills, anatomy/dissection tutorials, physiology/pharmacology laboratory practical sessions, clinical placements and completed eLearning materials. This learning served as their pre-work.

**Fig. 1:**
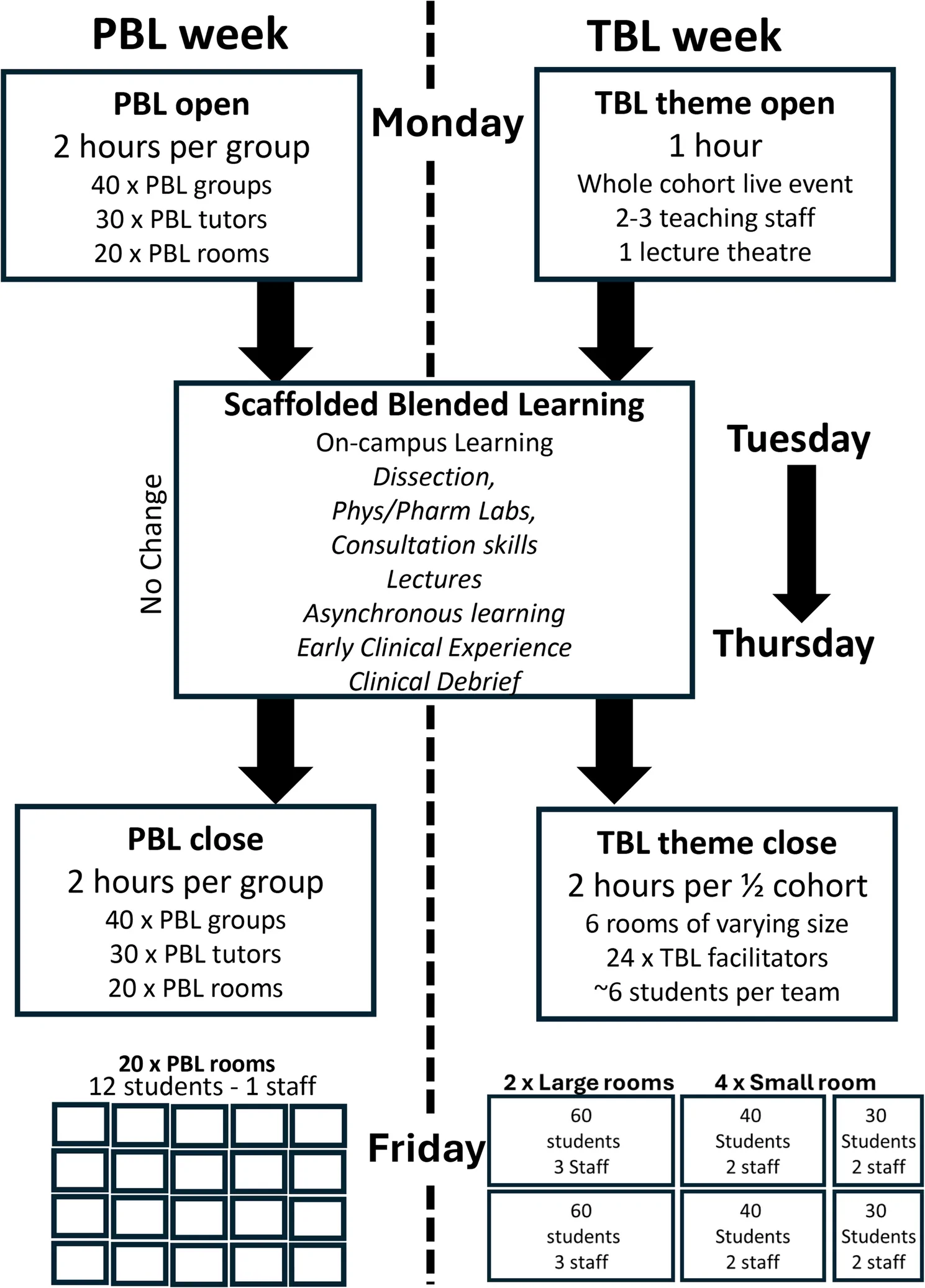
A TBL themed weekly structure is less staff time intensive whilst maintaining the core learning structure. The diagram illustrates the flow of teaching activities across the week. On Monday, students begin with *PBL open* sessions or a *TBL theme open* session. Throughout the week, learning is supported by *Scaffolded Blended Learning*, including on-campus and other asynchronous content. At the end of the week, students complete *PBL close* sessions or *TBL theme close* sessions

Each week culminated in a two-hour mandatory structured TBL session (iRAT, tRAT, application exercises, expert panel - see Fig. 1). Sessions concluded with an expert panel, UoM’s second adaptation to the standard TBL model, during which clinical experts and basic scientists addressed student questions and clarified key concepts. Three times throughout the semester, students also undertook a peer-review activity, where they evaluated both themselves and other members of their TBL team. Students were asked to answer three questions about each team member: (1) “This team member was well-prepared for their learning” (rated on a rubric from strongly disagree to strongly agree), (2) “This team member contributed well to the team” (rated on a rubric from strongly disagree to strongly agree); and (3) “How did this student contribute to the team?” (an open-text comment requiring a minimum response of 6 words). The peer review was formative, did not force discrimination (i.e. it did not involve ranking), and it was used primarily for the personal development of the students and to ensure no unhealthy team dynamics were developing. TBL for the students was an entirely formative learning experience with no summative component [45].

The cohort of ~ 440 was split into two groups, and each of the two sessions took place across six classrooms that were interconnected and linked via a live online broadcast. Two of our TBL classrooms were classified as large rooms (hosting 60 students; 10 TBL teams), while 4 were small rooms hosting 24–36 students or 4–6 TBL teams (see Fig. 1). Within their rooms, students worked in their TBL teams of 5–6 students on their TBL activities for the week, tailored to that theme’s ILOs, while 2–3 academic staff members facilitated the session and led intra-room discussions.

TBL learning materials were authored and delivered in the Learning Activity Management (LAMs) platform [46], which recorded item‑level responses, timestamps and extensive session metadata (for example, room allocation and cohort split).

The analysis presented in this paper was centred on the 2023/2024 (435 students) and 2024/2025 (426 students) Year 1 MBChB cohorts. The 2023/2024 cohort was the first to receive the TBL curriculum and represented our implementation year. At that stage, the concept of using performance analytics to guide early intervention had not yet been developed and was therefore introduced only in the following academic year. The 2024/2025 cohort was the first in which learning analytics were used to trigger bespoke, targeted support mid-semester. All enrolled students participating in the themed learning weeks and the timetabled TBL sessions were eligible for inclusion. The size and organisational complexity of the programme are comparable to other high-volume medical schools, providing a context in which findings may inform large-cohort delivery more broadly.

### Data sources and measures

To achieve a holistic overview of TBL implementation, data were derived from multiple sources. Operational data (staffing, rooms) were extracted from timetabling and estates records. Student experience data, which included quantitative ratings and qualitative free text comments, were obtained from end-of-semester evaluation surveys (administered to all students at the end of the last TBL session of each semester). The PBL session student feedback was from the teaching year 2021/22, which represented the last internal evaluation data of PBL.

Data on iRAT and tRAT performance (see [47, 48]) was obtained from Learning Analytics exported from the LAMs system. Peer and self-assessment ratings were collected via a criterion-referenced rubric which was administered three times during the semester (after Theme 2, Theme 4 and Theme 7). Summative outcome data were end-of-semester MCQ examinations.

### Student experience analysis and Net positivity calculations

Students were asked at the end of each semester to complete a survey focused on their experiences of TBL (see Table 1). This data was obtained in classroom via an anonymous survey in both semester 1 (385 students/89% response rate) and semester 2 (224 students/52% response rate). The majority of survey items used a five-point Likert scale. Students were presented with a statement (e.g. “I would rate the TBL sessions as excellent”) and asked to select a response ranging from Strongly Disagree (1) to Strongly Agree (5). For analysis, responses of “Agree” and “Strongly Agree” were grouped as positive responses, while “Disagree” and “Strongly Disagree” were grouped as negative responses. Positive and negative response rates were calculated as the percentage of total responses falling within these categories.

**Table 1:** Survey questions: A complete list of the questions asked to students through mentimeter

**List of survey questions**
Overall I would rate TBL sessions this semester as excellent
The TBL sessions have helped identify gaps in my learning
The mix of different application exercises has been appropriate
The TBL facilitators were good in guiding discussion in my room
The expert panel was able to clarify parts of my learning
Overall, I am satisfied with my Semester learning experience
Overall I would rate this semester as well organised
Overall each week's content had good alignment with the learning objectives of the theme

To provide a balanced measure of overall sentiment, a “net positivity” score was also calculated using the formula:

$$\mathrm{Net}\;\mathrm{positivity}=\mathrm{Positive}\;\mathrm{response}\;\mathrm{percentage}\;\!{-}\;\mathrm{Negative}\;\mathrm{response}\;\mathrm{percentage}$$

For example, if 65% of students responded positively to a statement and 21% responded negatively, the resulting net positivity score would be + 44% (65% − 21% = 44%). This indicates that positive responses substantially outweighed negative responses. This approach produces a score ranging from − 100% (all responses negative) to + 100% (all responses positive), with values above zero indicating that positive responses outweighed negative responses.

Open response questions asked what students enjoyed and what could be improved, and responses were analysed using a structured thematic approach. All analysis was conducted using pandas, re, and matplotlib libraries in Python.

### Performance metrics and environmental neutrality

iRAT mean scores per student per theme were the primary performance indicator. Content sensitivity was tested by comparing iRAT distributions across themes (one-way ANOVA with Tukey post‑hoc). Environmental neutrality was examined by comparing morning (09:00–11:00) versus midday (11:00–13:00) classes (Welch’s t-tests) and small versus large rooms defined a priori by capacity (Welch’s t-tests). These analyses were planned to differentiate content-driven variance from scheduling or locational effects. In addition to evaluating student performance, theme-level analyses were intended to explore whether readiness data could serve as a programme-level indicator of curriculum alignment and relative content difficulty.

### Predictive validity of readiness metrics

Criterion validity was assessed by regressing end-of-semester test scores on iRAT averages under multiple scopes using linear regression. Using all themes in Semester 2 and all themes in Semester 1, we estimated R² as variance explained by readiness. Early-warning utility was tested by restricting iRAT to Themes 1–4 and repeating regressions. Stability across cohorts was examined by replicating analyses in both 2023 and 2024.

### Peer and self-assessment

Peer assessment used a rubric aligned to TBL contribution and readiness. Students rated teammates and themselves after Themes 2, 4, and 7. We analysed (i) iRAT distributions by performance decile, (ii) the association between peer and self-ratings, and (iii) the association between peer ratings and iRAT. Between-group differences were evaluated (one‑way ANOVA with Tukey post‑hoc). This analysis was designed to examine convergent validity between behavioural (peer) and cognitive (iRAT) measures as an indicator of the reliability of peer assessment within the programme.

### Analytics triggered intervention

In 2024 (the first year of analytics-enabled support), mid-semester intervention was implemented after Theme 4. Eligibility was defined a priori as the bottom 50 students by mean iRAT across Themes 1–4. The Academic Advisors of these 50 students were notified, and they met individually with flagged students. Each student then received a bespoke support package tailored to their specific needs. While the contents varied by individual, packages typically included a combination of the following: targeted study-skills coaching; library led sessions on evidence search and resource use; referral to the Disability Advisory and Support Service (DASS) for screening or adjustments; counselling referral where appropriate; and structured mentorship from an academic or senior student to help scaffold accountability and sustained engagement.

The success of the intervention was evaluated by comparing the 50 students’ mean iRAT score for Themes 1–4 (pre) versus Themes 5–7 (post) using paired t-tests. For historical comparison, the same analysis was conducted in 2023 (first TBL year without analytics-triggered support). End-of-semester performance was compared between the at-risk subgroup in 2023 and 2024 and between whole-cohort means to monitor for cohort-wide grade inflation. To examine collaborative amplification, we compared iRAT and tRAT across themes at cohort level, expecting tRAT to exceed iRAT.

### Statistics and missing data

Missing iRAT data (that is, as a result of student absences) were not imputed in primary analyses; sensitivity checks with single-item mean imputation for students missing one iRAT in Themes 1–4 yielded similar early-warning results. Students withdrawing before semester end were excluded from summative analyses but were retained in descriptive summaries where relevant.

All statistical analyses and figures were produced in GraphPad Prism [49]. Tests were two‑tailed with α = 0.05. For multiple comparisons, Tukey corrections were applied as appropriate. Linear regression outputs included R² and 95% CIs; non‑parametric alternatives (Wilcoxon signed‑rank; Spearman’s ρ) were used as sensitivity checks where assumptions were not met.

### Ethics

Ethical approval to study the transition from PBL to TBL was approved by the University of Manchester’s Proportionate Research Ethics Committee (2024-18448-32454). Routinely collected educational and feedback data were analysed under the University’s quality-enhancement remit (ethical exemption code: 2026-26901-48359). Data were pseudonymised prior to analysis and reported in aggregate. Processing complied with institutional data-protection policy and UK GDPR. Students were informed at induction that anonymised performance and engagement data may be used to improve teaching and support; opt-out was available for entry of free text comments.

## Results

### Efficiency and structure: TBL reduces both the infrastructure and manpower burdens of PBL delivery model

To address the challenge of delivering medical education at scale, we analysed the structural requirements of PBL versus TBL (Fig. 1). In the PBL structure, each weekly cycle comprises a two-hour opening and a two-hour closing session per group of ~ 12 students, requiring approximately 30 tutors and 20 rooms to accommodate 40 groups. This equates to around 80 h of staff time per each open and close, giving a weekly total of 160 staff hours and 80 room bookings. This aligns with sector-wide observations that PBL’s logistical demands become increasingly difficult to sustain as cohort sizes expand [20, 23, 25].

By contrast, TBL consolidates activities into a one‑hour whole‑cohort opening session supported by three staff members (equating to three hours of staff time) in one lecture theatre and duplicate two-hour half cohort TBL sessions each supported by 14 facilitators across 6 rooms (56 facilitator hours and 13 room bookings). In total, weekly delivery of TBL requires 59 staff hours and 13 rooms, compared with 160 staff hours and 80 rooms for PBL; this equates to a reduction of roughly 63% in staff time and an impressive 84% reduction in room utilisation, a crucial factor that reduced pressure on oversubscribed teaching spaces. All other elements of teaching (lectures, dissection, physiology/pharmacology laboratories, clinical skills etc.) remain constant between models. Thus, human and room resource gains stem from delivery architecture rather than diminution of learning opportunities, consistent with constructive alignment principles [50] and with TBL guidance for large cohorts [31, 33, 34].

Moreover, whereas PBL necessitated a large pool of tutors with limited ownership of materials and whose expertise did not always align with the subject matter [20], the TBL model allows for a smaller group of facilitators, many of whom are also directly involved in the design of learning resources. Staff allocation to TBL sessions is determined by disciplinary speciality and interest in the pedagogy, which has fostered stronger ownership and repaired this weakness of PBL. These changes demonstrate that TBL offers a more sustainable and scalable model for delivering large cohort medical education. This remodelling of weekly teaching motivated further evaluation of whether student experience was maintained and whether the revised format yielded additional educational benefits. Given that staffing availability and teaching space are common constraints across medical schools, these structural efficiencies have potential relevance for other institutions managing expansion or resource pressure.

### Student experience was not altered by the change to TBL

**Fig. 2:**
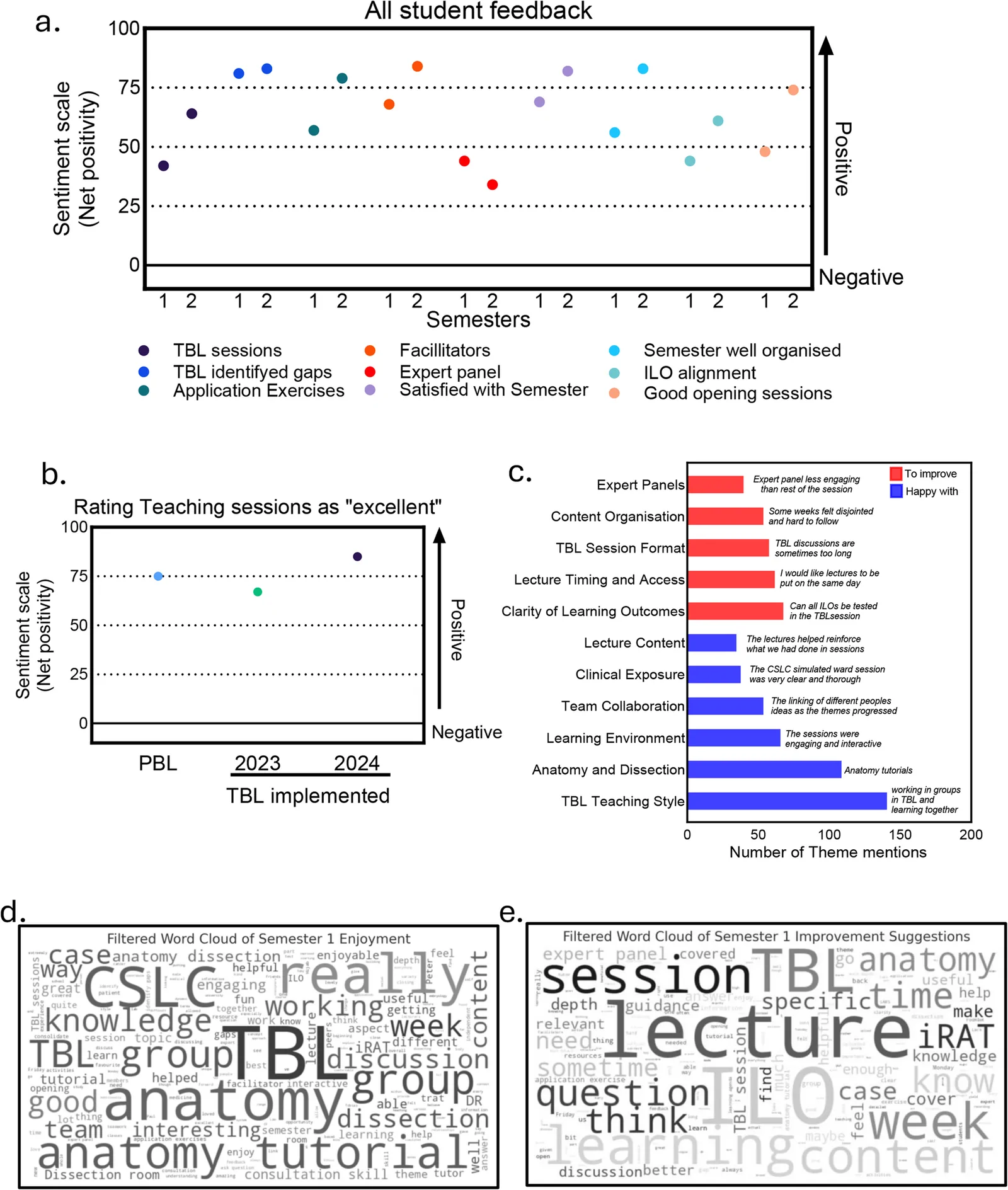
A TBL themed week is well received comparable to PBL, with high performance in identifying students learning gaps.** A** Net sentiment levels across key teaching activities as asked by individual questions (See Table 1). Each point represents all positive scores minus negative scores. Lowest *n* = 219/438. **B** Comparative student feedback of “Overall, I would rate TBL/PBL sessions this semester as excellent:” for PBL 2021 (*n* = 72), TBL 2023 (*n* = 104), TBL 2024 (*n* = 385). **C** Bar charts comparing the proportion of students reporting positive (red) and negative (blue) perceptions of major curriculum components. This was generated using open responses analysed thematically to “What are the aspects of Semester x have you enjoyed the most?” (*n* = 318) or “Of the things that you did not enjoy during Semester X (if any), what ideas do you have for how they may be improved??” (*n* = 286). **D** Filtered word cloud of free-text comments highlighting themes associated with student enjoyment. Prominent terms include *TBL*, *anatomy*, *CSL*, *group*, and *tutorial*. **E** Filtered word cloud of student suggestions for improvement, with frequently mentioned themes such as *lecture*, *IRAT*, *content*, *week*, and *questions*

To determine whether structural gains compromised student experience, we tracked net positivity scores across both semesters (Fig. 2A), where scores above zero indicate that positive responses outweighed negative responses. Student feedback following the adoption of TBL was consistently positive across all evaluated aspects of the pedagogy. The statement “TBL sessions help me identify gaps in my week’s learning” achieved particularly strong responses, with net positivity scores exceeding 78% in both semesters. UoM adaptations to incorporate TBL into the established teaching structures were also viewed favourably by students. Whole-cohort opening sessions achieved net positivity scores greater than 48%, indicating that positive responses substantially outweighed negative responses. Similarly, the online expert panel involving both basic science and clinical experts achieved net positivity scores exceeding 40%, demonstrating an overall favourable student perception of this addition to the pedagogy (Fig. 1, Fig. 2A).

Compared with PBL, the proportion of students rating learning sessions as “excellent” showed a slight decline during 2023, the first year of TBL implementation (2023). This short-term decline is consistent with the “implementation dip” described by Fullan [51], reflecting the adjustment period typical of major educational changes [52]. Subsequently, student ratings of sessions as “excellent” increased in the following year (2024), ultimately exceeding the pre-implementation level (Fig. 2B).

Open response questions asked what students enjoyed and what could be improved. Analysis of these responses revealed positive themes clustering around TBL and teamwork, anatomy sessions, clinical skills teaching, and lecture content (Fig. 2C). Students highlighted that the readiness assurance tests and application exercises revealed gaps in their knowledge (Fig. 2A and D), reflecting literature on the testing effect and feedback for learning [44, 53, 54]. Negative feedback focused primarily on lecture coherence and the alignment of TBL with intended learning outcomes (Fig. 2C). Indeed, when “TBL” was identified in negative contexts, much of the criticism related to ILO alignment with the sessions (Fig. 2D-E). This supports existing research that active learning activities must be clearly mapped to the curricular to ensure continued student engagement [55, 56]. 

### iRAT “Readiness” Metrics are indicative of theme week alignment, have structural neutrality and are predictive of student end of semester performance

**Fig. 3:**
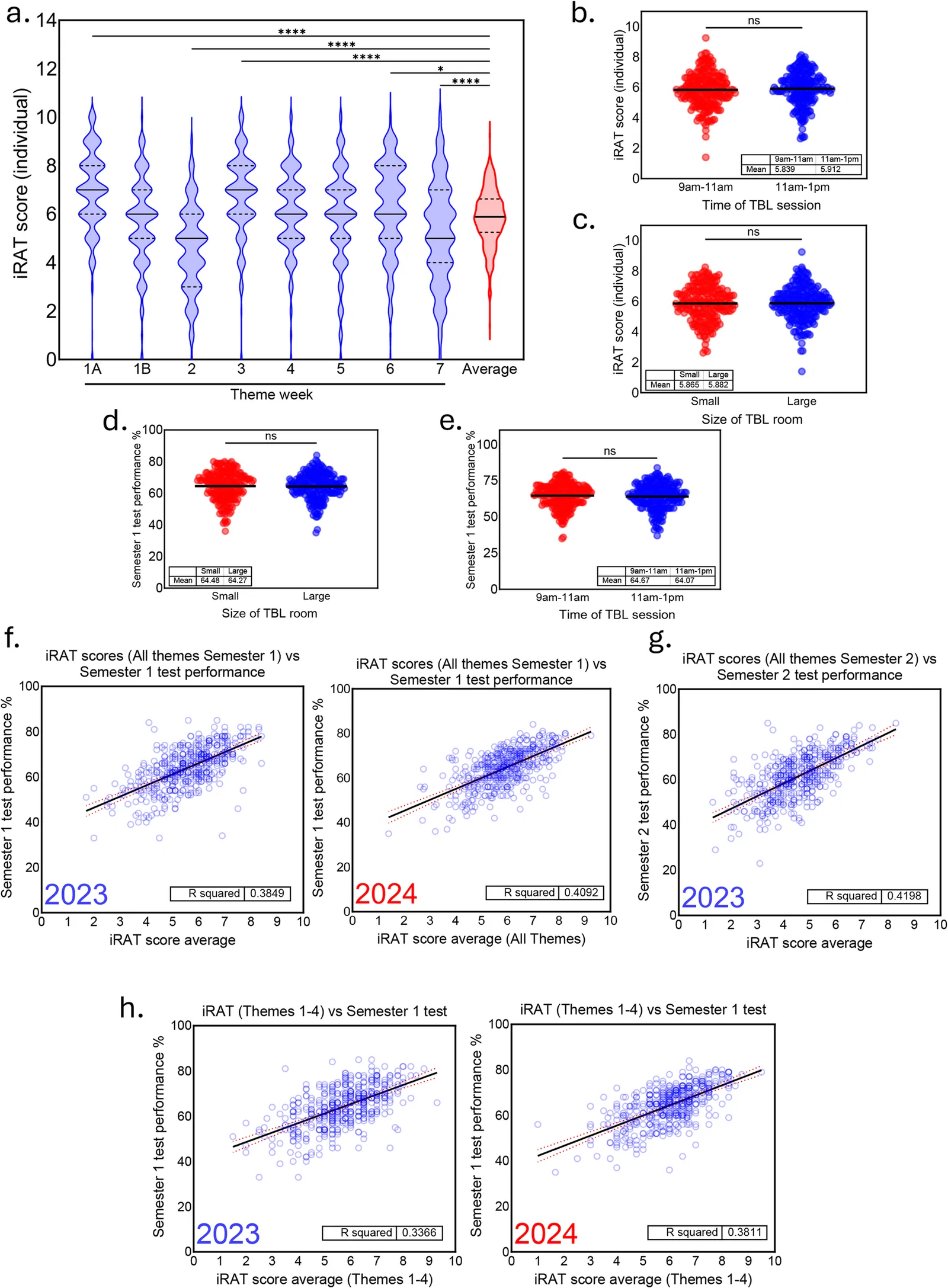
A TBL session and individual scores are impacted by the theme of the week and correlates with test performance.** A** Violin plots showing the distribution of individual readiness assurance test (iRAT) scores across all themes compared to the average of all themes. Solid lines represent median values, statistical analysis used One-way ANOVA with Dunnets- all themes compared to the average. Theme 1 A (*n* = 411), Theme 1B (*n* = 406), Theme 2 (*n* = 402) Theme 3 (*n* = 379), Theme 4 (*n* = 409), Theme 5 (*n* = 403), Theme 6 (*n* = 411) Theme 7 (*n* = 398). **B** Individual data point plots comparing iRAT performance for Early TBL session (red, *n* = 215) and Late (blue, *n* = 213). Line represents the mean values, statistical analysis used Welchs corrected two tailed unpaired T-Test. **C** Individual data point plots comparing iRAT performance for small room (red, *n* = 209) and large room (blue, *n* = 219). Line represents the mean values, statistical analysis used Welchs corrected two tailed unpaired T-Test. **D** Individual data point plots comparing Semester test performance for small room (red, *n* = 209) and large room (blue, *n* = 219). Line represents the mean values, statistical analysis used Welchs corrected two tailed unpaired T-Test. **E** Individual data point plots comparing Semester test performance for Early (red, *n* = 215) and Late (blue, *n* = 213). Line represents the mean values, statistical analysis used Welchs corrected two tailed unpaired T-Test. **F** Scatterplots with regression lines comparing semester 1 average iRAT score vs. end of semester 1 test score in both 2023 (*n* = 434) and 2024 (*n* = 433) cohorts. Each point represents a student. **G** Scatterplot with regression lines comparing semester 2 average iRAT score all themes vs. end of semester 2 test score in 2023 (*n* = 434) cohort. Each point represents a student. **H** Scatterplots with regression lines comparing semester 1 average iRAT score for the first 4 themes vs. end of semester 1 test score in 2023 (*n* = 434), 2024 (*n* = 433) cohorts. Each point represents a student

To identify whether the iRAT captures meaningful variance in knowledge acquisition, we compared performance across the different medical themes that comprise the curriculum (Fig. 3A). We found that iRAT scores for the cohort vary significantly between themes (Supplemental Fig. 1), suggesting that performance levels are influenced by content difficulty and the degree of alignment between the weekly materials, ILOs, and assessment coverage, rather than by random fluctuation. This pattern indicates that readiness data can function as a real-time curriculum signal, enabling identification of themes where learning materials, workload, or assessment alignment may require review.

When team performance was considered (Supplemental Fig. 1), students consistently scored higher on the tRAT than on the iRAT across all themes. This is an expected finding that reflects the benefits of peer explanation, team accountability, and collaborative reasoning in consolidating understanding in TBL [31, 33]. To exclude environmental artefacts, we tested two hypotheses. First, because time of day can influence cognitive performance [57–59] we examined student performance in the iRAT in the 9–11am versus 11–1pm sessions (Fig. 3B); we found no difference, indicating temporal neutrality. Second, given debates about class size effects on engagement [60–63], we compared iRAT performance in small versus large rooms (Fig. 3C). Again, we found no differences: this is consistent with evidence that TBL supports interaction in large cohorts via structured accountability [7, 30]. Consistent findings were obtained when these factors were compared against end-of-semester test performance (Figs. 3D–E), confirming that neither room size nor session timing influenced summative outcomes. Similarly, we observed that gender had no effect on performance (Supplemental Fig. 2). Interestingly, students born outside the UK achieved lower iRAT scores than home students, yet this difference did not persist in the final semester test (Supplemental Fig. 2). Although this observation warrants further investigation, it may suggest that TBL’s collaborative structure helps consolidate understanding and close early gaps in knowledge.

Having ruled out the most logistical and easily measurable confounders, the predictive criterion validity of the iRAT was tested. That is, we investigated how well early readiness scores forecast later summative achievement by correlating iRAT scores with end-of-semester summative exam performance. using all themes in Semester 1 of 2023 (Fig. 3F; R²≈0.42), Semester 2 of 2023 (Fig. 3G; R²≈0.38) and Semester 1 of 2024 (Fig. 3F; R²≈0.41). Intriguingly, Semester 1 iRAT performance correlated strongly with Semester 2 iRAT performance and Semester 2 end-of-semester test results (Supplemental Fig. 3). These consistent associations indicate that iRAT performance remains stable across semesters and predicts subsequent achievement, supporting its longitudinal reliability. Collectively, these findings suggest that the iRAT captures a general underlying learning ability rather than any theme-specific performance differences (Figs. 2E and 3A; Supplemental Fig. 3). Given that research has highlighted that early academic intervention is more effective [64–66], we next sought to determine how early the iRAT can predict end-of-semester test performance. Restricting the analysis to the first four themes of Semester 1 showed that iRAT performance by Theme 4 already correlated strongly with end-of-semester test outcomes in both 2023 (R² ≈ 0.34) and 2024 (R² ≈ 0.38), indicating that meaningful predictive information is available early enough to support targeted action (Fig. 3H). Together, these findings position the iRAT as a content‑sensitive, structurally robust, and criterion‑valid indicator suitable for analytics‑driven support. These characteristics suggest that readiness-based indicators could form the basis of early-warning systems in other programmes using structured active-learning approaches.

### Student performance in the early themes is normally distributed, and peer review can simultaneously identify the same tails

**Fig. 4:**
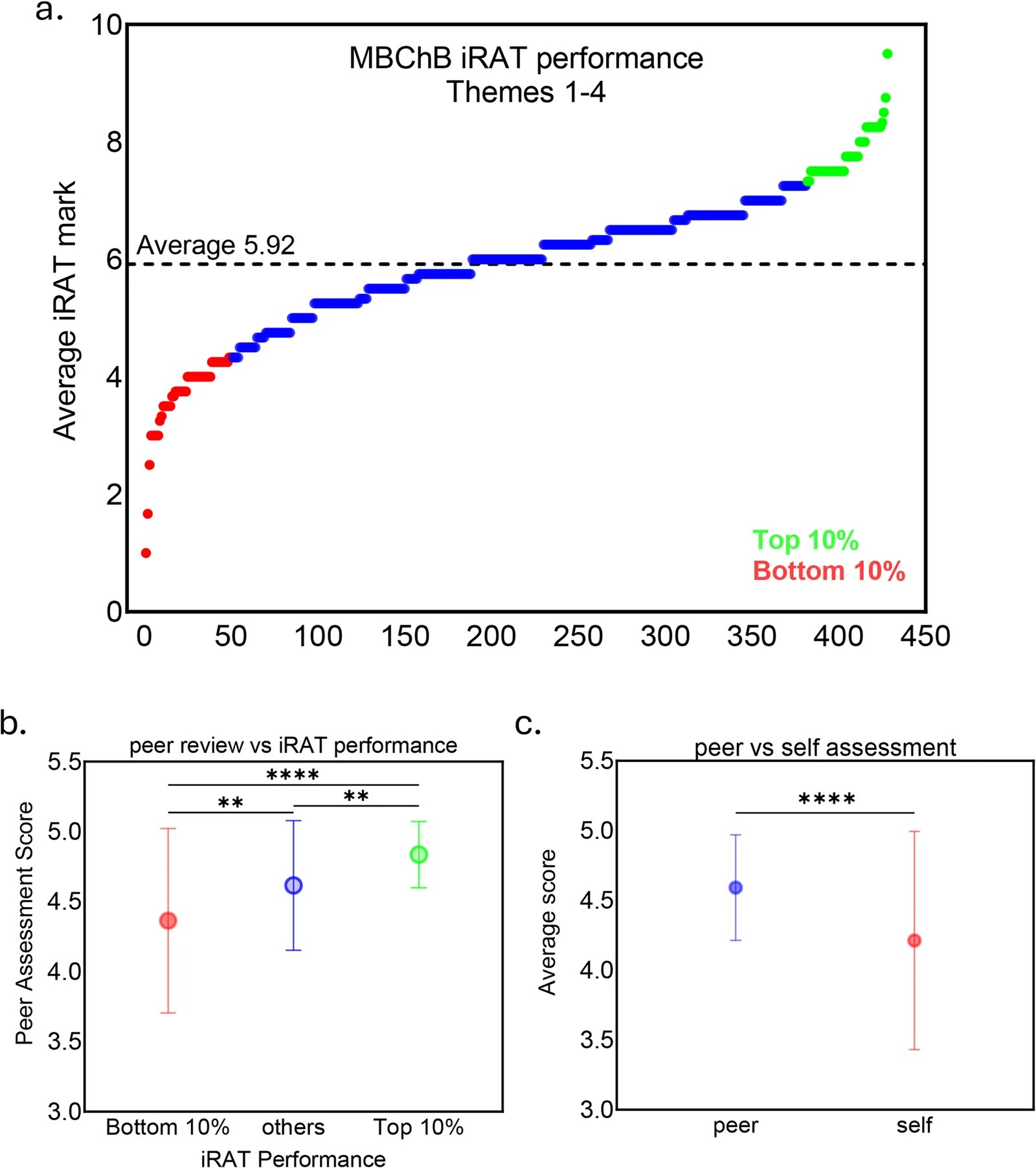
Peer review also identifies struggling students early **A** Individual students' average iRAT score in the first 4 themes, ranked in order visualising the bottom and top 10% of performance and the average of the 2024 cohort (*n* = 428). **B **Mean average iRAT score in the first 4 themes separated into the bottom (*n* = 45) and top 10% (*n* = 49) and the remaining cohort (*n* = 333). Dots indicate the mean value with S.D. statistical analysis used One-way ANOVA with Tukey. **C** Mean of the peer (*n* = 427) and self (*n* = 402) assessments scores. Dots indicate the mean value with S.D. statistical analysis used two-tailed T-Test

To complement the identification of theme 1–4 as an early indicator of student performance, we identified the highest and lowest performing tails (Fig. 4A). As peer evaluation is an essential element to successful TBL implementation [67–70] we tested whether peer feedback was successful in identifying the same relative strengths as the iRAT. Students in the lowest iRAT decile received significantly lower peer ratings during themes 1–4 (Fig. 4B), suggesting that under‑preparation is visible to teammates. Indeed, grouping by performance bands showed significant peer‑rating differences between the top and bottom deciles when mapped to the iRAT (Fig. 4B). These results confirm that peer assessment, when criterion‑referenced and transparent, yields valid judgments of contribution and professionalism [68, 71–73]. The convergence between peer ratings and independent performance metrics provides programme-level evidence that peer evaluation is functioning as intended, supporting its use as a quality-assured behavioural assessment rather than solely a reflective activity.

For medical curricula, this behavioural lens aligns evaluation with competency‑based medical education (CBME) frameworks emphasising teamwork, accountability and entrustment [74, 75]. An important caveat to that is that medical student’s self‑assessment scores were significantly lower than scores given by peers (Fig. 4C), indicating that their metacognitive awareness is low so we must complement this data with external quantitative performance metrics. For this reason, we solely deployed iRAT readiness as an early indicator of at-risk students; however, an amalgam of quantitative (iRAT) and behavioural analytics (peer review score) provides a potential multidimensional early‑warning system to be explored in the future. 

### Early TBL analytics combined with bespoke intervention improves semester learning performance and decreases risk of failing

**Fig. 5:**
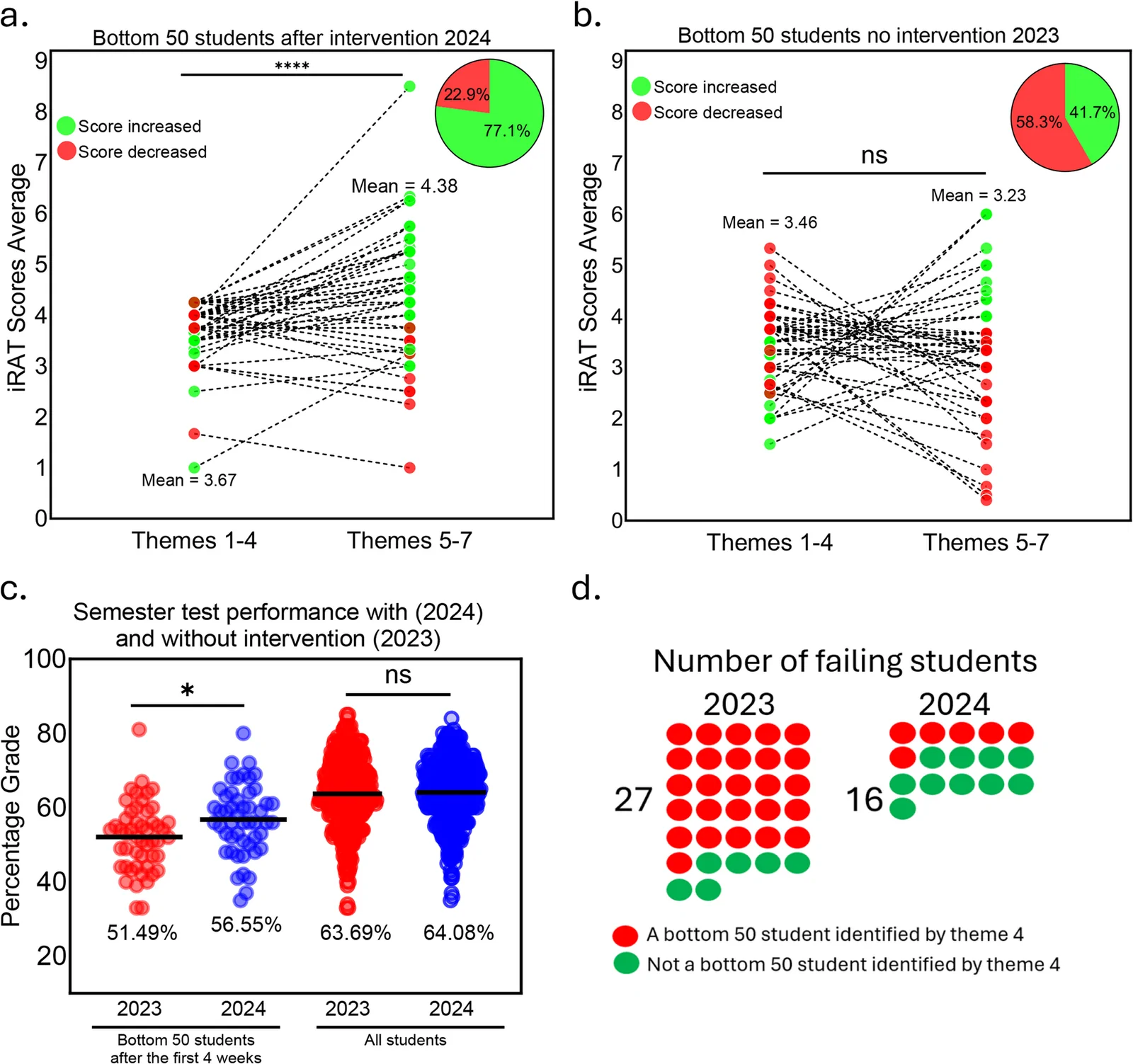
Early identification of struggling students leads to better test outcomes and reduction in exclusion **A** Individual students average iRAT score in the first 4 themes and the average iRAT score for the final 4 themes, each dot represents an individual student, green indicates improvement and red indicates declined score linked with a line for the 2024 cohort (*n* = 50). Pie chart showing spread of improvers and decliners, statistical analysis was a two tailed paired T-Test. **B** Individual students average iRAT score in the first 4 themes and the average iRAT score for the final 4 themes, each dot represents an individual student, green indicates improvement and red indicates declined score linked with a line for the 2023 cohort (*n* = 50). Pie chart showing spread of improvers and decliners, statistical analysis was a two tailed paired T-Test. **C** Individual data point plots comparing Semester test performance for identified bottom 50 students after theme 4 for 2023 (*n* = 50) and 2024 (*n* = 50) and the Semester test performance for the whole cohort in 2023 (*n* = 434) and 2024 (*n* = 434). Line represents the mean values, statistical analysis used Welchs corrected two tailed unpaired T-Tests. **D** Dot graphic identifying the spread of the students that failed the Semester test in 2023 and 2024. Colour indicates if the student was identified as a bottom 50 student after the first 4 themes (red) or not (green)

Having established predictive and behavioural signals, we examined whether acting on them alters trajectories. After theme 4, the bottom 50 students’ Academic Advisors received performance alerts for underperforming students and provided bespoke support packages, which included academic advising, study skills workshops, targeted library support, Disability Advisory and Support Service (DASS) referrals, counselling, and mentorship. The exact support package was on a case-by-case basis and not prescriptive. Among the bottom 50 students identified, the mean iRAT score increased from 3.67 (themes 1–4) to 4.38 (themes 5–7) with intervention (*p* < 0.0001) (Fig. 5A), whereas the matched 2023 cohort without intervention showed no improvement, and, in fact, performance decreased throughout the semester (Fig. 5B). Among the bottom 50 students, only 41% improved their iRAT scores in the latter half of the semester in 2023, whereas with intervention in 2024 this proportion rose to 77%. This indicates that the improvement was broadly distributed across the group rather than driven by a few isolated high performers (Figs. 5A–B). End-of-semester test outcomes reflected the same pattern: students who received intervention in 2024 achieved a mean score of 56%, compared with 51% for the equivalent non-intervention cohort in 2023, representing a significant improvement. Importantly, whole cohort mean scores remained stable across years (Fig. 5C).

The overall number of students failing the end-of-semester test fell from 27 to 16 with early intervention (Fig. 5D). Importantly, and the most indicative evidence of the power of early intervention, is the observation that the number of students in the bottom 50 that failed the semester fell from 22 to 6, a 72% reduction (Fig. 5D). Taken together, these findings exemplify constructive programmatic assessment in action: frequent, low-stakes assessments generating evidence to enable quick and proportionate intervention [43, 44] consistent with learning-analytics frameworks that emphasise data-driven support for students [76–79].

## Discussion

This study provides evidence from a large operational medical programme that a structured TBL model can address challenges widely faced across medical education, including expansion of cohort size, resource constraints, and the need for early support of struggling learners. Importantly, this study does not seek to demonstrate the effectiveness of TBL itself, which is already well established, but rather to examine how the routine data generated within TBL can be used to support programme-level educational decision-making.

The structural redesign allowed for a reduction in demands on staff time and estates space without impacting the quality of education, addressing the challenges of educating at scale faced by medical schools [1, 4]. Sustained satisfaction and relatively stable “excellent” ratings indicate that quality was, broadly, maintained during change, consistent with research showing active learning reduces failure and improves performance across disciplines [80] and with reviews of TBL specifically in health professions education [26, 30, 31].

Moreover, the analytics generated within TBL provided us with a theoretically grounded approach to identify students who were at risk of under-performing. Readiness measures were content-sensitive (Fig. 3A), robust to environmental variation (Figs. 3B–E), and demonstrated criterion validity against summative outcomes (Figs. 3F–H).

Three distinct programme-level functions emerged from these analyses. First, early iRAT performance provided a reliable early-warning signal that enabled targeted, personalised intervention for students at risk. Second, variation in mean performance across themes offered a continuous indicator of curriculum alignment and relative content difficulty, supporting ongoing quality assurance and refinement. Third, the strong association between peer ratings and independent readiness measures provided convergent evidence that peer evaluation was functioning as a valid behavioural assessment mechanism. Together, these findings position TBL data not only as a student assessment tool but as a source of curriculum intelligence.

The observed content sensitivity of iRAT performance aligns with constructivist perspectives on learning, which propose that students build new understanding by actively integrating incoming information with existing cognitive frameworks or prior knowledge [81–83]. The theme-specific variation seen here suggests that readiness is not uniform but shaped by how effectively preparatory materials connect with, and extend, these prior schemata.

The invariance to time‑of‑day and class size mitigates equity concerns associated with timetabling or estate constraints [60, 57, 61, 58]. The predictive relationships align with the testing effect and formative‑feedback literatures, in which frequent retrieval and high‑quality feedback enhance long‑term retention and transfer [53, 54]. Peer ratings provided convergent behavioural evidence aligned with CBME and professional activities [74, 75], strengthening confidence that flagged learners face both cognitive and professional development risks.

The interventions were deliberately bespoke. Rather than provide a one‑size‑fits‑all workshop, advisors were informed of current under‑performance and signposted students to targeted supports matched to need: study skills development; structured library sessions; DASS referrals for formal adjustments; counselling when wellbeing barriers were evident; and mentorship for accountability and social support. This design responds to evidence that multi‑component, personalised approaches yield stronger gains than generic remediation [84, 85]. This aligns with the growing emphasis on actionable interventions in the learning analytics literature [86–88]. The observed improvements in both readiness and summative performance for the bottom 50 students, alongside stable cohort means (Figs. 5 A–5 C), therefore, represent an equity uplift rather than grade inflation, addressing institutional imperatives around differential attainment [3, 10].

The results are broadly consistent with existing evidence that TBL maintains or enhances student satisfaction while improving engagement and knowledge retention in medical education [26, 30, 31]. The brief decline in satisfaction during the first year of implementation parallels earlier reports of an initial “implementation dip” as students adapt to new expectations [51], followed by recovery once familiarity and facilitation quality stabilise. This suggests that the transitional challenges observed at UoM are not unique, but characteristic of major pedagogical reform.

The present study extends prior work in three ways. While previous research has demonstrated the educational benefits of TBL and the formative value of readiness assurance, few studies have shown how these routinely collected data can be operationalised at scale to (1) trigger real-time student support (2), inform continuous curriculum quality monitoring, and (3) provide evidence for the validity of behavioural assessments such as peer review.

Two broader implications emerge from this work. First, embedding analytics within an active-learning curriculum operationalises the principles of programmatic assessment: triangulating evidence from multiple sources, supporting proportional decision-making, and fostering meaningful feedback cycles [43, 44]. Second, these findings demonstrate that TBL extends beyond pedagogy: when implemented at scale, it functions as an embedded analytics infrastructure, transforming routine teaching activity into a continuous source of evidence for student support, curriculum governance, and quality assurance for large medical programmes.

Although conducted within a single institution, several features support the transferability of the findings. The educational challenges addressed (growing cohort sizes, limited staffing and teaching space, and concerns about differential attainment) are widely reported across medical schools nationally and internationally. The intervention did not rely on unique institutional resources, but rather on standard elements of TBL (readiness assurance, structured teams) combined with routine learning analytics and existing academic advising structures. These components are available to many programmes, suggesting that the approach represents a scalable design strategy rather than a context-specific innovation.

For medical educators, this has direct relevance to the long-standing challenge of identifying and supporting struggling learners before summative failure occurs. The TBL framework provides an evidence-based, scalable approach to linking performance analytics with human oversight, maintaining educational standards while promoting equity and wellbeing.

### Strengths and weaknesses of the study

A key strength of this study lies in its scale and ecological validity. Conducted within the largest medical school in the United Kingdom, the evaluation captured full-cohort data from two successive intakes, encompassing over 850 students across two years of curriculum reform. The use of a consistent digital platform (LAMs) ensured standardisation of delivery and data capture, allowing for reliable comparison between themes, environments, and cohorts. Integration of operational, survey, and performance data provided a triangulated perspective on educational and institutional outcomes.

The study focuses on system-level analytic processes rather than the effectiveness of a specific teaching method, supporting the broader relevance of the findings to programmes already using TBL or other structured active-learning approaches.

However, several limitations must be acknowledged. The bespoke nature of the intervention introduces heterogeneity in delivery. Students received differing combinations of study-skills sessions, DASS referrals, library support, and mentorship, without fidelity monitoring, making it difficult to isolate which components drove improvement. Advisor and facilitator effects may also have contributed, as variations in advising style or experience were not modelled. A Hawthorne or expectancy effect also cannot be excluded: being made aware that they were underperforming may have caused students to modify their behaviour independently of the intervention itself.

The single-institution design may limit contextual generalisability. However, the large cohort size, full-programme implementation, and replication across consecutive years enhance external validity. The study evaluates system-level processes rather than institution-specific content, supporting the relevance of the findings to other large medical programmes.

### Future research

Future research should investigate the bespoke support packages provided to each at-risk student to investigate the impacts and effects of each components. This will clarify which elements drive the greatest performance gains and where resources can be most efficiently targeted. It would also be valuable to evaluate the cost-effectiveness of the broader TBL curriculum transformation itself, particularly in terms of staffing and space utilisation, as sustainable adoption in large medical programmes depends on demonstrable efficiency as well as educational impact. In parallel, predictive models could be enhanced by incorporating additional data (specifically engagement analytics and attendance) alongside iRAT and peer-assessment measures, potentially using interpretable machine-learning approaches. Longitudinal follow‑up into clinical placements could test transfer from preclinical analytics‑guided remediation to workplace performance, addressing calls to connect curricular innovation to downstream competence [89–91].

## Conclusions

In summary, this study demonstrates how routine data generated within large-scale TBL can be leveraged as a programme-level analytics system to support early intervention, continuous curriculum monitoring, and quality assurance of behavioural assessment. It aligns pedagogical design with programmatic assessment and learning analytics, translating frequent low-stakes evidence into timely, proportionate support that demonstrably changes trajectories. As medical schools internationally seek to expand capacity while maintaining quality, integrating structured active learning with embedded analytics and personalised support offers a practical and scalable model for improving performance, equity, and student experience at scale.

## Supplementary Information

Supplementary Material 1.

Supplementary Material 2: Supplemental Figure 1 associated to Figure 3 - Student teams have enhanced performance in all themes. Violin plots showing the distribution of individual readiness assurance test (iRAT blue) scores across all themes compared and team readiness assurance test (tRAT red) scores across all themes . Solid lines represent median values, statistical analysis used iRAT - Theme 1A (n=411), Theme 1B (n=406), Theme 2 (n=402) Theme 3 (n=379), Theme 4 (n=409), Theme 5 (n=403), Theme 6 (n= 411) Theme 7 (n= 398) All tRAT n=40.

Supplementary Material 3: Supplemental Figure 2 to Figure 3 -Sex and place of birth do not alter Semester test performance and place of birth does not affect semester test performance but does effect iRAT performance that does not improve over time.A) Individual data point plots comparing iRAT or Semester test performance for UK registered (red, n=333) and Elswhere register (blue, n=101). Line represents the mean values, statistical analysis used Welchs corrected two tailed unpaired T-Test. B) Individual data point plots comparing iRAT performance for male (red, n=177) and large room (blue, n=256). Line represents the mean values, statistical analysis used Welchs corrected two tailed unpaired T-Test.

Supplementary Material 4: Supplemental Figure 3 to Figure 3 - iRAT scores of both semesters correlate with each other and semester 1 performance correlates with semester 2 performance A) Scatterplot with regression lines comparing semester 1 average iRAT score all themes vs semester 2 average iRAT score all themes in 2023 (n=434) cohort. Each point represents a student. B) Scatterplot with regression lines comparing semester 1 average iRAT score all themes vs end of semester 2 test score in 2023 (n=434) cohort. Each point represents a student.

## Data Availability

All data generated or analysed during this study are included in this published article and its supplementary information files.

## Abbreviations

AE: Application Exercise
ANOVA: Analysis of Variance
CBME: Competency–Based Medical Education
CI: Confidence Interval
DASS: Disability Advisory and Support Service
ILO: Intended Learning Outcome
iRAT: Individual Readiness Assurance Test
LAMs: Learning Activity Management System
MBChB: Bachelor of Medicine, Bachelor of Surgery
MCQ: Multiple Choice Question
NHS: National Health Service
PBL: Problem–Based Learning
R²: Coefficient of Determination
tRAT: Team Readiness Assurance Test
TBL: Team–Based Learning
UoM: University of Manchester
